# Effect of low tidal volume ventilation on lung function and inflammation in mice

**DOI:** 10.1186/1471-2466-10-21

**Published:** 2010-04-21

**Authors:** Hans P Hauber, Dörte Karp, Torsten Goldmann, Ekkehard Vollmer, Peter Zabel

**Affiliations:** 1 Division of Pathophysiology of Inflammation, Research Center Borstel, Borstel, Germany; 2 Medical Clinic, Research Center Borstel, Borstel, Germany; 3 Division of Experimental Pathology, Research Center Borstel, Borstel, Germany

## Abstract

**Background:**

A large number of studies have investigated the effects of high tidal volume ventilation in mouse models. In contrast data on very short term effects of low tidal volume ventilation are sparse. Therefore we investigated the functional and structural effects of low tidal volume ventilation in mice.

**Methods:**

38 Male C57/Bl6 mice were ventilated with different tidal volumes (Vt 5, 7, and 10 ml/kg) without or with application of PEEP (2 cm H_2_O). Four spontaneously breathing animals served as controls. Oxygen saturation and pulse rate were monitored. Lung function was measured every 5 min for at least 30 min. Afterwards lungs were removed and histological sections were stained for measurement of infiltration with polymorphonuclear leukocytes (PMN). Moreover, mRNA expression of macrophage inflammatory protein (MIP)-2 and tumor necrosis factor (TNF)α in the lungs was quantified using real time PCR.

**Results:**

Oxygen saturation did not change significantly over time of ventilation in all groups (P > 0.05). Pulse rate dropped in all groups without PEEP during mechanical ventilation. In contrast, in the groups with PEEP pulse rate increased over time. These effects were not statistically significant (P > 0.05). Tissue damping (G) and tissue elastance (H) were significantly increased in all groups after 30 min of ventilation (P < 0.05). Only the group with a Vt of 10 ml/kg and PEEP did not show a significant increase in H (P > 0.05). Mechanical ventilation significantly increased infiltration of the lungs with PMN (P < 0.05). Expression of MIP-2 was significantly induced by mechanical ventilation in all groups (P < 0.05). MIP-2 mRNA expression was lowest in the group with a Vt of 10 ml/kg + PEEP.

**Conclusions:**

Our data show that very short term mechanical ventilation with lower tidal volumes than 10 ml/kg did not reduce inflammation additionally. Formation of atelectasis and inadequate oxygenation with very low tidal volumes may be important factors. Application of PEEP attenuated inflammation.

## Background

Years ago it was recognized in animal models that mechanical ventilation with high tidal volumes can cause alveolar disruption and pulmonary edema [1]. However, it was not until the ARDS (adult respiratory distress syndrome) network trial that it became clear that mechanical ventilation plays an important role in the propagation of lung injury, a process referred to as ventilator-induced lung injury (VILI) [2,3]. A large number of animal studies have investigated the effects of mechanical ventilation with high tidal volumes compared to lower tidal volumes. These studies expanded our knowledge on the mechanisms of VILI [4]. Excessive alveolar distension can cause lung injury due to increased vascular permeability, alteration in lung mechanics, and increased production of inflammatory mediators [5-9]. On the other hand lower tidal volumes can be associated with progressive alveolar derecruitment leading to atelectasis [10]. This may be prevented by use of deep inflation and positive end-expiratory pressure (PEEP) [11].

In humans a tidal volume of 6 ml/kg is considered to be protective at least in ARDS. In murine models tidal volumes of 10 ml/kg are currently used as normal or protective ventilation to compare the effects of high tidal and low tidal volume ventilation [12-15]. This volume lies within the tidal volume of spontaneously breathing mice [16,17]. However, information on the use of lower tidal volumes e. g. 6 ml/kg is sparse. Since this volume is close to that of spontaneously breathing animals it may induce less damage.

We therefore aim to investigate the effects of lower tidal volumes (5 and 7 ml/kg) compared to the commonly used tidal volume of 10 ml/kg without and with addition of PEEP and compared to spontaneously breathing animals in a mouse model of mechanical ventilation. We used physiological measurements to describe functional changes. Moreover, we measured neutrophilic inflammation and inflammatory cytokine expression. Our first goal of this study was to expand the knowledge of functional and structural changes due to mechanical ventilation with very low tidal volumes in a mouse model. The second goal was to determine whether lower tidal volumes may be less injurious than the commonly used tidal volume of 10 ml/kg. These data may help to understand lung function and inflammation at very low tidal volumes and in a second step may improve protective ventilation strategies.

## Methods

### Animal preparation

Experiments were carried out in accordance with the Animal Protection Law of Germany. All experiments were approved by the local Ethics Committee. 10- to 12-week old male C57/Bl6 mice (Charles River Labs, Berlin, Germany) (25-35 g) were anesthetized by intraperitoneal injection of ketamine (1%) and xylazin (0.02%). Additional anesthetic was given throughout the protocol as needed. After tracheostomy with a secured 18-gauge metal cannula mechanical ventilation was initiated using a flexivent (Scireq, Montreal, Canada) computer-controlled small animal ventilator. Oxygen saturation and pulse rate were monitored using the MouseOx oximeter (Starr Life Science, Pittburgh, PA). The sensor was placed on the back leg along the leg axis. The mice were covered throughout the experiments to maintain body temperature.

### Protocols

After anaesthesia mice were randomized to mechanical ventilation with different tidal volumes (Vt: 5 ml/kg, 7 ml/kg, and 10 ml/kg bodyweight) without and with application of 2 cm H_2_O of positive end-expiratory pressure (PEEP). All mice were ventilated with a frequency of 150/min and a FI0_2 _of 0.21 (normal air). Mechanical ventilation was carried out for at least 30 min until a drop in oxygen saturation of > 20% or a pulse rate below 200/min. In detail the ventilation groups were as follows: Vt 5 ml/kg without PEEP (n = 7), Vt 7 ml/kg without PEEP (n = 7), Vt 10 ml/kg without PEEP (n = 6), Vt 5 ml/kg + PEEP (n = 5), Vt 7 ml/kg + PEEP (n = 6), Vt 10 ml/kg + PEEP (n = 7). Four spontaneously breathing mice served as normal controls (n = 4). At the end of each experiment the lung of each animal was divided into two parts. One part was immediately snap frozen in liquid nitrogen for later RNA extraction. The other part was put into formalin for later histological examination.

### Lung function measurements

At the beginning and at the end of mechanical ventilation a TLC (total lung capacity) manoeuvre was performed. With this manoeuvre lungs are inflated with a pressure of up to 30 cm H_2_O for a total of six seconds in order to standardize the volume history and to prevent preexisting atelectasis. During mechanical ventilation lung function measurements were performed every 5 min using the flexivent ventilator. Resistance (Newtonian resistance, Rn), tissue damping (G), and tissue elastance (H) were determined with forced manoeuvres on the basis of a constant phase model of the lungs as described previously [18].

### Histological measurements

Lungs were inflated for histological analysis with a pressure of up to30 cm H_2_O for a total of six seconds. Formalin fixed tissue specimens were sectioned and stained with hematoxilin eosion (HE) according to standard protocols. Numbers of polymorphonuclear leukocytes (PMN) were counted per high powered field by two independent observers in a blinded fashion. The within observer coefficient of variation was less than 5%.

### RNA extraction and reverse transcription

RNA from whole lung tissue samples was extracted using an RNeasy Mini Kit (Qiagen). Reverse transcription was performed with 0.5-1.0 μg of RNA per reaction using Superscript II reverse transcriptase (RT, 200 U per reaction) (Invitrogen) and oligo-dT in the presence of an RNase inhibitor (RNase Out, Invitrogen). The RNA was reverse transcribed in 30 μl of total volume at 65°C for 10 min, at 42°C for 60 min, and at 100°C for 1 min. The resultant first-strand complementary DNA (cDNA) was used as template for PCR.

### Quantitative real time PCR (QRTPCR)

QRTPCR was carried out using a LightCycler system (Roche Diagnostics, Mannheim, Germany). Macrophage inflammatory protein (MIP)-2 mRNA, tumor necrosis factor (TNF)α mRNA and hypoxanthine phosphoribosyltransferase (HPRT) mRNA expression was quantified using QRTPCR. HPRT was used as house keeping gene. Primers were based on published mRNA sequences and were designed to span at least two exons in order to avoid binding to genomic DNA. Specific amplification using these primers was confirmed by ethidium bromide staining of the predicted size of the PCR products on an agarose gel. PCR was performed using the QuantiTect SYBR Green PCR Kit (Qiagen) with the appropiate primers and samples according to the manufacturer's protocol. In brief, 1 μl of cDNA was added to 10 μl of 2× QuantiTect SYBR Green PCR master mix, 8 μl of RNase-free water, and 0.5 μl of each primer (20 μM) resulting in a total volume of 20 μl. All PCR experiments were carried out in triplicate.

### Statistics

Comparisons were made between the different experimental groups. In each group values at the beginning and at the end of ventilation (after 30 min) were compared. An overall ANOVA, followed by multiple testing with the Bonferroni correction, was performed. Differences between conditions were assessed by means of post hoc pairwise comparison with the Dunnet test. A P value of less than 0.05 was considered statistically significant. All values are given as means ± SEM if not otherwise stated.

## Results

### Hemodynamic parameters

**Figure 1:**
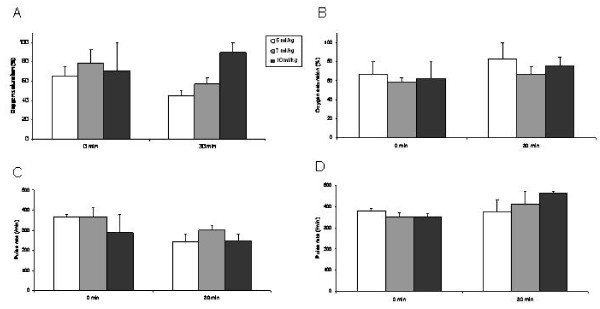
**Oxygen saturation (A, B) and pulse rate (C, D) at baseline (0 min) and after 30 min of mechanical ventilation with different tidal volumes without (A, C) and with addition of PEEP (B, D)**. Mean values ± SEM.

Oxygen saturation showed no significant difference at baseline (0 min) between the groups regardless whether PEEP was applied or not (P > 0.05) (Fig 1A, B). There was a decrease in oxygen saturation after 30 min of mechanical ventilation in the groups that were ventilated with low tidal volumes (5 ml/kg and 7 ml/kg) without PEEP but this was not statistically significant (P > 0.05) (Fig 1A). In contrast, oxygen saturation increased or remained stable in the other groups. However, no statistically significance was observed (P > 0.05) (Fig 1A, B).

There was no significant difference in the pulse rate at baseline (0 min) between the different groups regardless whether PEEP was applied (Fig 1D) or not (Fig 1C). Pulse rate decreased in the groups that were ventilated without addition of PEEP (Fig 1C). In contrast, in the groups that were ventilated with addition of PEEP pulse rate increased (Fig 1D). However, these effects were not statistically significant (P > 0.05).

### Lung function measurements

**Figure 2:**
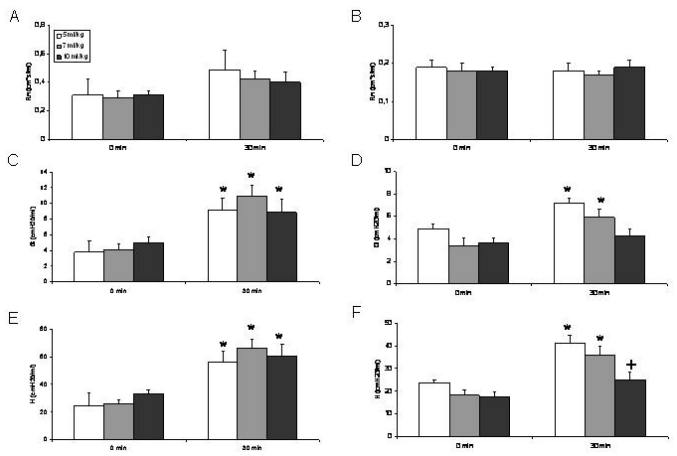
**Resistance (Rn) (A, B), tissue damping (G) (C, D), and tissue elastance (H) (E, F) at baseline (0 min) and after 30 min of mechanical ventilation with different tidal volumes without (A, C, E) and with addition of PEEP (B, D, F)**. *: P < 0.05 vs baseline. +: P < 0.05 vs all other groups. Mean values ± SEM.

At baseline (0 min) no significant difference was measured for Rn (Newtonian resistance), G (tissue damping), and H (tissue elastance) between the different groups (Fig 2).

Rn increased after 30 min of ventilation in the groups without PEEP (Fig 2A) whereas it remained almost stable in the groups that were ventilated with addition of PEEP (Fig 2B). A significant increase (1.6-fold) in Rn was only observed in the group with a Vt of 5 ml/kg without addition of PEEP (P < 0.05).

Tissue damping (G) significantly increased after 30 min in all groups that were ventilated without addition of PEEP (Vt 5 ml/kg: 2.4-fold; Vt 7 ml/kg: 2.7-fold; Vt 10 ml/kg: 1.8-fold) (P < 0.05) (Fig 2C). In the groups that were ventilated with addition of PEEP a significant increase was observed in the groups with a Vt of 5 ml/kg (1.5-fold) and with a Vt of 7 ml/kg (1.7-fold) (P < 0.05) (Fig 2D). In contrast, no significant change was observed in the group that was ventilated with a Vt of 10 ml/kg and PEEP (P < 0.05).

There was a significant increase in tissue elastance (H) after 30 min in the groups that were ventilated without addition of PEEP (Vt 5 ml/kg: 2.3-fold; Vt 7 ml/kg: 2.6-fold; Vt 10 ml/kg: 1.8-fold) (P < 0.05) (Fig 2E). In the groups that were ventilated with addition of PEEP a significant increase of H was observed for the groups with a Vt of 5 ml/kg (1.7-fold) and with a Vt of 7 ml/kg (1.9-fold) (P < 0.05) but not for the group with a Vt of 10 ml/kg (P > 0.05) (Fig 2F). In addition, H after 30 min of ventilation was significantly lower in the group with a Vt of 10 ml/kg compared to all other groups that were ventilated with addition of PEEP (P < 0.05) (Fig 2F).

### Quantification of polymorphonuclear leukocytes in the lungs

**Figure 3:**
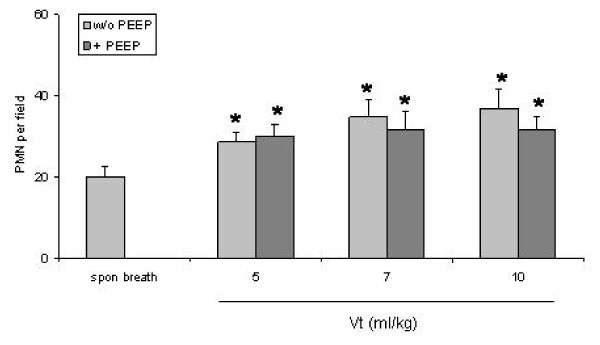
**Infiltration of lung parenchyma with polymorphonuclear leukocytes (PMN)**. Columns represent mean+SEM of spontaneous breathing (spon breath) mice and ventilation groups. *: P < 0.05 vs spontaneous breathing animals.

**Figure 4:**
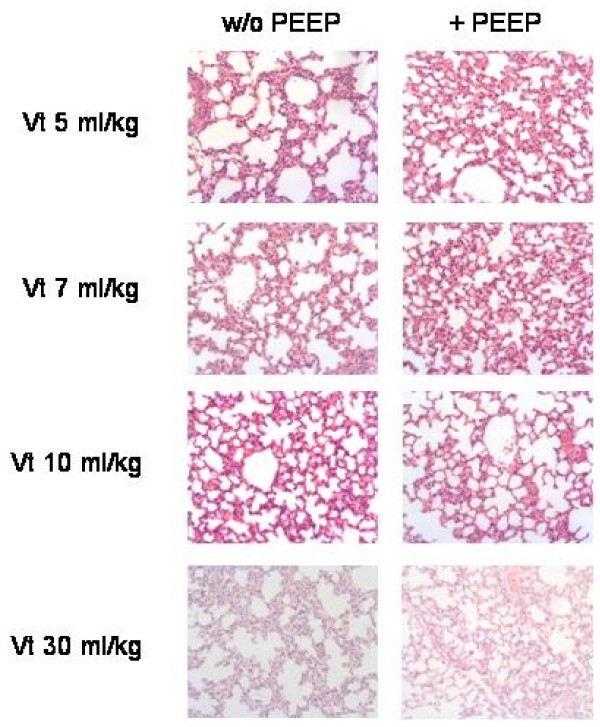
**Histological sections of lung parenchyma after mechanical ventilation with different tidal volumes without and with application of PEEP**. HE staining. Original magnification ×400.

Ventilation without PEEP significantly increased the numbers of polymorphonuclear leukocytes (PMN) at all used tidal volumes (Vt 5 ml/kg: 28.7 ± 2.4 cells/field, Vt 7 ml/kg: 34.9 ± 4.3 cells/field, and Vt 10 ml/kg: 36.8 ± 4.9 cells/field) compared to spontaneously breathing mice (20.0 ± 2.5 cells/field) (P < 0.05) (Fig 3). With addition of PEEP the numbers of PMN were reduced but were still significantly increased in ventilated mice (Vt 5 ml/kg: 25.5 ± 0.6 cells/field, Vt 7 ml/kg: 26.8 ± 2.1 cells/field, and Vt 10 ml/kg: 31.7 ± 3.2 cells/field) compared to spontaneously breathing mice (P < 0.05). Fig 4 shows original histology section stained with HE of the eight different ventilation groups. Infiltration of intraalveolar septae with PMN is noted with all applied ventilation protocols.

### Expression of MIP-2 and TNFα

**Figure 5:**
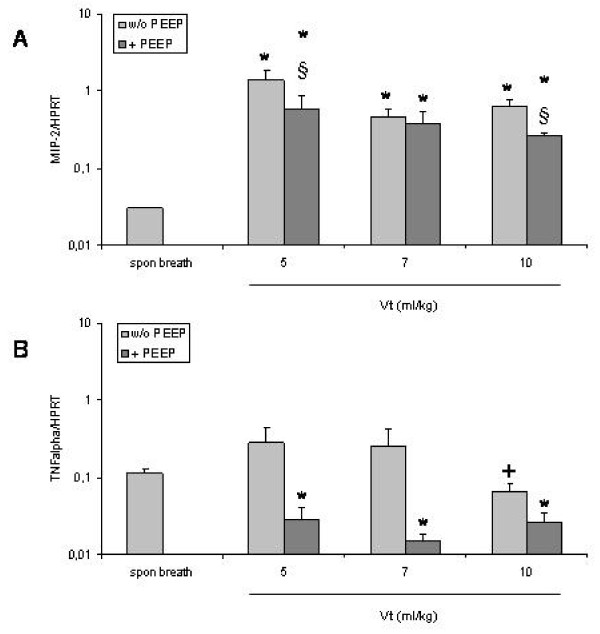
**Expression of MIP-2 (A) and TNFα (B) mRNA in the lungs of mice that were ventilated with different tidal volumes without and with application of PEEP**. Columns represent mean+SEM of spontaneous breathing (spon breath) mice and ventilation groups. *: P < 0.05 vs spontaneous breathing animals. § P < 0.05 vs + PEEP. +: P < 0.05 vs ventilation with a Vt of 5 ml/kg or 7 ml/kg without PEEP.

Mechanical ventilation with different tidal volumes (Vt 5, 7, and 10 ml/kg) significantly increased MIP-2 mRNA expression compared to spontaneously breathing mice regardless whether PEEP was applied (49-fold, 19-fold, and 12-fold, respectively) or not (46-fold, 15-fold, and 21-fold, respectively) (P < 0.05) (Fig 5A).

Addition of PEEP significantly reduced MIP-2 mRNA expression at tidal volumes 5 and 10 ml/kg (P < 0.05). MIP-2 gene expression was significantly decreased with a Vt of 10 ml/kg and PEEP compared to a Vt of 5 ml/kg without or with PEEP (P < 0.05) (Fig. 5A). While mechanical ventilation with tidal volumes of 5 ml/kg and 7 ml/kg increased TNFα mRNA expression (2-fold) ventilation with a tidal volume of 10 ml/kg decreased TNFα gene expression by about 50% compared to spontaneously breathing mice (Fig 5B). These effects were not statistically significant (P > 0.05) but TNFα gene expression was significantly lower with a Vt of 10 ml/kg compared to a Vt of 5 ml/kg and 7 ml/kg (P < 0.05). In contrast, mechanical ventilation with PEEP significantly reduced TNFα mRNA expression at all used tidal volumes (5-fold, 10-fold, and 5-fold, respectively) (P < 0.05) (Fig 5B).

## Discussion

In the present study we found that mechanical ventilation with lower tidal volumes than 10 ml/kg and the loss of PEEP were associated with worse lung function and increased expression of inflammatory mediators in a murine model.

A large number of studies have used high tidal volumes (20-30 ml/kg) to investigate the effect of excessive alveolar distension and ventilator induced lung injury in mouse models. Those studies demonstrated that high tidal volumes can induce inflammation, barrier disruption, and pulmonary edema [1,4-9,12-15]. On the other hand low tidal volumes used in protective ventilation may lead to atelectasis and hypoxia [10]. Data on the effect of very small tidal volumes in mechanical ventilation in murine models are sparse. In our study we sought out to investigate the effect of tidal volumes as low as recommended by the ARDS network study as being protective (6 ml/kg; [2]). This is below the tidal volume of 10 ml/kg that is commonly used as reference or protective ventilation in mouse models to compare the effects of low and high tidal ventilation [12-15] but it is in the range of normal tidal volumes of spontaneously breathing animals estimated on the basis of data from plethysmographic studies [16,17]. Our aim was to specifically examine changes in hemodynamic parameters, lung function measurements and inflammatory cytokine expression in order to evaluate whether lower tidal volumes would be less injurious. Moreover, we sought to expand the knowledge on ventilator-induced changes in mouse lungs with low tidal volumes.

Lungs of mice and humans are different in terms of size, structure, and functional behaviour. The normal frequency of breathing is much higher mice (up to 200 breaths/min) compared to humans. Minute ventilation mainly depends on frequency since tidal volumes are small (5 to 15 ml/kg; [16,17]). Therefore it was possible that ventilation with very small tidal volumes resulted in inadequate minute ventilation volumes causing hypoxia. On the other hand data from the ARDSnet demonstrated that ventilation close to physiologic parameters is the least injurious [2]. However, we did not perform experiments with different respiratory rates which is a limitation of the study.

In the present study low tidal volumes were associated with very low oxygen saturation during mechanical ventilation. The most possible reason for this is that small tidal volumes (5 and 7 ml/kg) did not recruit sufficient alveolar units for adequate oxygenation. However, the tidal volumes we used are in the range of tidal volumes of spontaneously breathing mice [16,17]. Moreover, there was no significant difference compared to a Vt of 10 ml/kg. Low tidal volumes may have caused atelectasis. Cycling opening and re-opening of the lung during the formation of atelectasis may cause increased shear forces thus leading to inflammation. Part of this effect could probably be prevented by application of PEEP. With the addition of PEEP oxygen saturation improved. Although we did not measure the lower inflection point it seems that the PEEP used in our study was high enough based on previous data from the literature [18]. However recent studies showed that higher PEEP values are not injurious to healthy mice [19,20].

With very small tidal volumes it is possible that gas exchange is hampered because of death space ventilation. Small volumes may not lead to adequate exchange of air in the alveoli if they are close to the death space. Although we cannot rule out the possibility that small tidal volumes led to hypoxia mostly due to death space ventilation we do not think that this was the main reason. First the mice used in our experiments were tracheotomized which minimizes anatomical death space. Second the system we used (Flexivent^®^) is constructed for the ventilation of small animals with small volumes. Death space in the system is very small.

In the present study the pulse rate did not significantly differ between the groups at baseline and after 30 min of ventilation. Although this is not a proof these data suggest that hemodynamics were not significantly altered by different tidal volumes. However, ventilation could be performed longer in the groups with a Vt of 10 ml/kg with or without PEEP compared to all other groups until criteria to stop ventilation were met (data not shown).

Resistance (Rn) increased during ventilation if no PEEP was applied but this effect was not statistically significant. In contrast Rn remained stable with the application of PEEP. However, Rn represents mostly the central airways. A significant increase in tissue damping (G) reflecting tissue resistance after 30 min of ventilation was observed. Our data agree with a study by Wilson and co-workers [21]. In that study resistance showed a significant increase with high tidal volume ventilation and a small not significant decrease with low tidal volume ventilation (9 ml/kg) [21].

We observed a significant increase in tissue elastance (H) after 30 min of ventilation. This has also been described in a study by Allen and co-workers [11]. In that study deep inflation has been shown to be beneficial when applied several times a minute. In our study we did not use deep inflation to recruit parts of the lung because we wanted to examine specifically the effect of small tidal volumes on lung function and cytokine expression without additional shear forces. Interestingly, tissue elastance was significantly lower after 30 min in the group with a Vt of 10 ml/kg and PEEP compared to all other groups.

Histological measurements revealed increased septal thickening in ventilated mice (data not shown). This finding most probably reflects formation of edema and influx of cells due to mechanical ventilation as been described in previous studies [22,23]. We observed a marked increase in the numbers of PMN in ventilated lungs compared to spontaneously breathing animals. Influx of PMN due mechanical ventilation has been described previously [24,25]. The application of PEEP had no significant effect on the numbers of PMN.

To further analyze the effect of low tidal volume ventilation on inflammation the expression of the neutrophil attractant cytokine MIP-2 and the proinflammatory cytokine TNFα was investigated. The increase in proinflammatory cytokine expression in groups with lower tidal volumes agree with previous data from the literature. Caruso and co-workers showed that ventilation with low tidal volumes caused similar proinflammatory and profibrogenic responses as high tidal volume ventilation compared to spontaneously breathing rats [26]. However, in that study the authors did not apply PEEP. In a recent study by Terragani and colleagues [27] low tidal volume ventilation was not associated with increased inflammation. However, in that study human patients were investigated and extracorporeal carbon dioxide removal was used. Agreeing with our data Nakos and co-workers demonstrated in increased numbers of PMN in the lungs of mechanically ventilated patients with atelectasis [28].

We observed a marked reduction of proinflammatory cytokine expression with the application of PEEP. Small tidal volumes may cause inflammation due to formation of atelectasis and increased shear forces during opening and re-opening of alveoli. It is not surprising that the application of PEEP that prevents the formation of atelectasis can reduce shear forces and thereby decrease expression of inflammatory mediators.

In our experiments mice were ventilated for 30 min. This has to be considered as a very short time frame. However, changes in lung function parameters, in neutrophil influx, and in cytokine expression could be observed compared to spontaneously breathing animals. Moreover, this time frame enabled us to apply different tidal volumes without additional oxygen to stabilize oxygen saturation. This minimizes a potential confounding effect of oxygen.

Oxygen saturation was very low in the animals with very small tidal volumes. Some of them were hypoxic or were exposed to severe hypoxia (oxygen saturation below 75% at baseline). Unfortunately, we did not perform blood gas analysis. However, inflammation was mostly increased with very low tidal volumes. Therefore hypoxia may not be the only cause for changes in inflammatory markers. We cannot clearly comment on survival issues because ventilation was terminated if there was a significant drop in oxygen saturation or reduced pulse rate prior to death of the animal. Based on the ventilation time in the different groups it has to be concluded that survival was probably shorter in the groups that were ventilated with either a Vt of wither 5 ml/kg or 7 ml/kg compared to the group with a Vt of 10 ml/kg regardless whether PEEP was applied or not.

We also looked at a group that was ventilated with a tidal volume of 30 ml/kg as a control for high-tidal ventilation. This group had elevated PMN numbers and significantly increased proinflammatory cytokine expression as expected from data from the literature (data not shown). Our data support the notion that both too low and too high volumes in mechanical ventilation lead to lung damage.

## Conclusion

In conclusion in the present study we demonstrated impaired oxygenation, lung function, and proinflammatory cytokine expression with lower tidal volumes than 10 ml/kg and the absence of PEEP in mechanically ventilated mice. Our data indicate that mechanical ventilation with lower tidal volumes than 10 ml/kg in mice are not less injurious. Whether atelectasis or impaired oxygenation play the major role remains to be further studied.

## Competing interests

The authors declare that they have no competing interests.

## Authors' contributions

HPH planned the study, performed experiments, analyzed the data and wrote the manuscript. DK, TG and EV performed the experiments. PZ analyzed and interpreted the data. All authors read and approved the final manuscript.

## Pre-publication history

The pre-publication history for this paper can be accessed here:

http://www.biomedcentral.com/1471-2466/10/21/prepub

## References

[B1] Webb HH Tierney DF Experimental pulmonary edema due to intermittent positive pressure ventilation with high inflation pressures: protection by positive end-expiratory pressure Am Rev Respir Dis 1974 110 556 565 4611290 10.1164/arrd.1974.110.5.556

[B2] The Acute Respiratory Distress Network Ventilation with lower tidal volumes as compared with traditional volumes for acute lung injury in the acute respiratory distress syndrome N Engl J Med 2000 342 1301 1308 10.1056/NEJM200005043421801 10793162

[B3] Tremblay LN Slutsky AS entilator-induced lung injury: from bench to bedside Intensive Care Med 2006 32 24 33 10.1007/s00134-005-2817-8 16231069

[B4] Frank JA Matthay MA Science review: mechanisms of ventilator-induced lung injury Crit Care 2003 7 233 241 10.1186/cc1829 12793874 PMC270664

[B5] Al-Jamal R Ludwig MS Changes in proteoglycans and lung tissue mechanics during excessive mechanical ventilation in rats Am J Physiol Lung Cell Mol Physiol 2001 281 L1078 L1087 11597898 10.1152/ajplung.2001.281.5.L1078

[B6] Corbridge TC Wood LD Crawford GP Chudoba MJ Yanos J Sznajder JI Adverse effects of large tidal volume and low PEEP in canine acid aspiration Am Rev Respir Dis 1990 142 311 315 2200314 10.1164/ajrccm/142.2.311

[B7] Dreyfuss D Basset G Soler P Saumon G Intermittent positive-pressure hyperventilation with high inflation pressures produces pulmonary mircovascular injury in rats Am Rev Respir Dis 1985 132 880 884 3901844 10.1164/arrd.1985.132.4.880

[B8] Uhlig S Ventilation-induced lung injury and mechanotransduction: stretching it too far? Am J Physiol Lung Cell Mol Physiol 2002 282 L892 L896 11943651 10.1152/ajplung.00124.2001

[B9] Veldhuizen RA Slutsky AS Joseph M McCraig L Effects of mechanical ventilation of isolated mouse lungs on surfactant and inflammatory cytokines Eur Respir J 2001 17 488 494 10.1183/09031936.01.17304880 11405530

[B10] Richard JC Maggiore SM Jonson B Mancebo J Lemaire F Brochard L Influence of tidal volume on alveolar recruitment. Respective role of PEEP and a recruitment maneuver Am J Respir Crit Care Med 2001 163 1609 1613 11401882 10.1164/ajrccm.163.7.2004215

[B11] Allen G Lundblad LK Parsons P Bates JH Transient mechanical benefits of a deep inflation in the injured mouse lung J Appl Physiol 2002 93 1709 1715 12381758 10.1152/japplphysiol.00473.2002

[B12] Altemeier WA Matute-Bello G Gharib SA Glenny RW Martin TR Liles WC Modulation of lipopolysaccharide-induced gene transcription and promotion of lung injury by mechanical ventilation J Immunol 2005 175 3369 3376 16116230 10.4049/jimmunol.175.5.3369

[B13] Chuimello D Pristine G Slutsky Mechanical ventilation affects local and systemic cytokines in an animal model of acute respiratory distress syndrome Am J Respir Crit Care Med 1999 160 109 116 10390387 10.1164/ajrccm.160.1.9803046

[B14] Wilson MR Choudhury S Goddard ME O'Dea KP Nicholson AG Takata M High tidal volume upregulates intrapulmonary cytokines in an in vivo model of ventilator-induced lung injury J Appl Physiol 2003 95 1385 1393 12807894 10.1152/japplphysiol.00213.2003

[B15] Wilson MR Choudhury S Takata M Pulmonary inflammation induced by high-stretch ventilation is mediated by tumor necrosis factor signalling in mice Am J Physiol Lung Cell Mol Physiol 2005 288 L599 L607 10.1152/ajplung.00304.2004 15489373

[B16] Tankersley CG Fitzgerald RS Kleeberger SR Differential control of ventilation among inbred strains of mice Am J Physiol Regul Integr Comp Physiol 1994 267 R1371 R1377 10.1152/ajpregu.1994.267.5.R1371 7977867

[B17] Tankersley CG Fitzgerald RS Levitt RC Mitzner WA Ewart SL Kleeberger SR Genetic control of differential baseline breathing pattern J Appl Physiol 1997 82 874 881 9074977 10.1152/jappl.1997.82.3.874

[B18] Bates JH Understanding lung tissue mechanics in terms of mathematical models Monaldi Arch Chest Dis 1993 73 134 139 8518775

[B19] Massa CB Allen GB Bates JHT Modeling the dynamics of recruitment and derecruitment in mice with acute lung injury J Appl Physiol 2008 105 1813 1821 10.1152/japplphysiol.90806.2008 18948446 PMC2612465

[B20] Cannizzaro V Berry LJ Nicholls PPK Zosky GR Turner DJ Hantos Z Sly PD Lung volume recruitment maneuvers and respiratory system mechanics in mechanically ventilated mice Respir Physiol Neurobiol 2009 169 243 251 10.1016/j.resp.2009.09.012 19788941

[B21] Wilson MR Choudhury S Goddard ME O'Dea KP Nicholson AG Takata M High tidal volume upregulates intrapulmonary cytokines in an in vivo mouse model of ventilator-induced lung injury J Appl Physiol 2003 95 1385 1393 12807894 10.1152/japplphysiol.00213.2003

[B22] Imanaka H Shimaoka M Matsuura N Nishimura M Ohta N Kiyono H Ventilator-induced lung injury is associated with neutrophil infiltration, macrophage activation, and TGF-beta 1 mRNA upregulation in rat lungs Anesthesia Analgesia 2001 92 428 436 10.1097/00000539-200102000-00029 11159246

[B23] Dreyfuss D Soler P Basset G Saumon G High inflation pressure pulmonary edema. Respective effects of high airway pressure, high tidal volume, and positive end-expiratory pressure Am Rev Respir Dis 1988 137 1159 1164 3057957 10.1164/ajrccm/137.5.1159

[B24] Sugiura M McCulloch PR Wren S Dawson RH Froese AB Ventilator pattern incluences neutrophil influx and activation in atelectasis-prone rabbit lung J Appl Physiol 1994 77 1355 1365 7836140 10.1152/jappl.1994.77.3.1355

[B25] Choudhury S Wilson MR Goddard ME O'Dea KP Takata M Mechanisms of early pulmonary neutrophil sequestration in ventilator-induced lung injury in mice Am J Physiol Lung Cell Mol Physiol 2004 287 L902 L910 10.1152/ajplung.00187.2004 15257987

[B26] Caruso P Meireles SI Reis LF Mauad T Martins MA Deheinzellin D Low tidal volume ventilation induces proinflammatory and profibrogenic response in lungs of rats Intensive Care Med 2003 29 1808 1811 10.1007/s00134-003-1908-7 12904859

[B27] Terragni PP Del Sorbo L Mascia L Urbino R Martin EL Birocco A Faggiano C Quintel M Gattinoni L Ranieri VM Tidal volume lower than 6 ml/kg enhances lung protection: role of extracorporeal carbon dioxide removal Anaesthesiology 2009 111 826 835 10.1097/ALN.0b013e3181b764d2 19741487

[B28] Nakos G Tsangaris H Liokatis S Kitsiouli E Lekka ME Ventilator-associated pneumonia and atelectasis: evaluation through bronchoalveolar lavage fluid analysis Intensive Care Med 2003 29 555 563 12595981 10.1007/s00134-003-1680-8

