# Correction: effect of low tidal ventilation on lung function and inflammation in mice

**DOI:** 10.1186/1471-2466-12-7

**Published:** 2012-02-23

**Authors:** Hans P Hauber, Dörte Karp, Torsten Goldmann, Ekkehard Vollmer, Peter Zabel

**Affiliations:** 1 Division of Pathophysiology of Inflammation, Research Center Borstel, Borstel, Germany; 2 Medical Clinic, Research Center Borstel, Borstel, Germany; 3 Division of Experimental Pathology, Research Center Borstel, Borstel, Germany

## Correction

In our publication "Effect of low tidal volume ventilation and inflammation in mice" [1] an error has occured in figure one. Due to technical limitations we were not able to measure oxygen saturation and heart rate in all animals. Unfortunately we only realized that we applied mean value and SEM when we re-checked our data because of a discussion with colleagues. We decided that it would not make sense to present mean values and the SEM because of the small numbers of animals in each group. We revised the figure (see revised Figure 1) and present now individual and mean values together with the numbers of animals that could be investigated. We apologize for any incovenience this may cause. However, the overall conclusions are not affected by this change.

**Figure 1:**
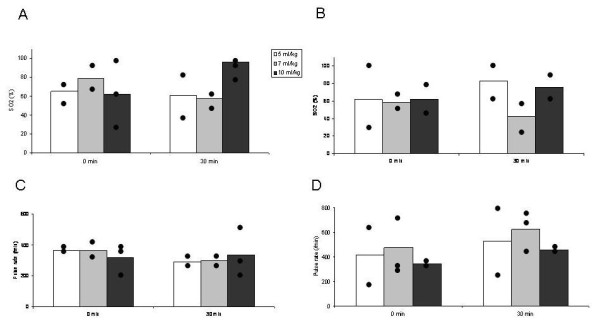
**Oxygen saturation (A, B) and pulse rate (C, D) immediately after the beginning of mechanical ventilation (0 min) and after 30 min (30 min) with different tidal volumes without (A, C) and with addition of PEEP (B, D)**. Bars represent mean values of 2 or 3 animals per group. Dots show individual values to demonstrate the high variability in measurements.

## Pre-publication history

The pre-publication history for this paper can be accessed here:

http://www.biomedcentral.com/1471-2466/12/7/prepub
